# Quantifying phase magnitudes of open‐source focused‐probe 4D‐STEM ptychography reconstructions

**DOI:** 10.1111/jmi.13409

**Published:** 2025-03-29

**Authors:** Toma Susi

**Affiliations:** ^1^ Faculty of Physics University of Vienna Vienna Austria

**Keywords:** 4D‐STEM, electron ptychography, focused‐probe, graphene, open‐source, phase quantification

## Abstract

Accurate computational ptychographic phase reconstructions are enabled by fast direct‐electron cameras with high dynamic ranges used for four‐dimensional scanning transmission electron microscopy (4D‐STEM). The availability of open software packages is making such analyses widely accessible, and especially when implemented in Python, easy to compare in terms of computational efficiency and reconstruction quality. In this contribution, I reconstruct atomic phase shifts from convergent‐beam electron diffraction maps of pristine monolayer graphene, which is an ideal dose‐robust uniform phase object, acquired on a Dectris ARINA detector installed in a Nion UltraSTEM 100 operated at 60 keV with a focused‐probe convergence semi‐angle of 34 mrad. For two different recorded maximum scattering angle settings, I compare a range of direct and iterative open‐source phase reconstruction algorithms, evaluating their computational efficiency and tolerance to reciprocal‐space binning and real‐space thinning of the data. The quality of the phase images is assessed by quantifying the variation of atomic phase shifts using a robust parameter‐based method, revealing an overall agreement with some notable differences in the absolute magnitudes and the variation of the phases. Although such variation is not a major issue when analysing data with many identical atoms, it does put limits on what level of precision can be relied upon for unique sites such as defects or dopants, which also tend to be more dose‐sensitive. Overall, these findings and the accompanying open data and code provide useful guidance for the sampling required for desired levels of phase precision, and suggest particular care is required when relying on electron ptychography for quantitative analyses of atomic‐scale electromagnetic properties.

## 1 INTRODUCTION

Aberration‐corrected scanning transmission electron microscopy (STEM) is a powerful tool for imaging^1^ and even manipulation^2^ of the atomic structure of materials, as well as the characterisation of their electronic and vibrational^3^ properties with electron energy‐loss spectroscopy. Its success has been spurred by rapid developments in instrumentation,^4^ understanding of irradiation damage,^5^ and advanced computational tools including machine learning.^6^ Recently, analysing all scattered electrons by recording 2D diffraction patterns from 2D scanned areas in a technique called 4D‐STEM has become increasingly powerful for virtual diffraction imaging, simultaneous resolving of light and heavy elements,^7^ and mapping of phase, orientation and strain as well as sample thickness and tilt.^8^ This is increasingly practical due to the commercialisation of fast and sensitive direct‐electron detectors,^9^, ^10^ which enable not only more accurate determination of the deflections of the electron probe,^11^ but also access to the redistribution of intensity within Bragg disks containing phase information.

Harnessing the redundancies in overlapping 4D datasets enables an efficient means of scanning coherent diffractive imaging called ptychography,^12^ where the complex electron probe and sample potentials can be reconstructed for post‐acquisition aberration correction^13^ and super‐resolution imaging,^14^ allowing sub‐Å projected spacings to be precisely measured in 2D heterostructures.^15^ Thin specimen act as phase objects, causing phase shifts of the electron waves that are directly correlated with atomic‐scale electromagnetic potentials. This has enabled the direct imaging of electrostatic potentials and the charge density^16^, ^17^, ^18^ as well as (at least) antiferromagnetic order.^19^, ^20^, ^21^ However, as the nuclei dominate electron scattering, it is extremely challenging to reliably tease out valence properties whose contribution is 10–100 times smaller, let alone the even weaker magnetic ones. Simulations based on first‐principles scattering potentials are vital^22^ to assist in the measurement of charge transfer,^23^, ^24^ but understanding optimal sampling conditions^25^, ^26^ and differences between algorithms,^27^ as well as quantifying the precision and accuracy of phase reconstructions, are pressing issues that have not been fully addressed yet.

Ptychographic reconstruction algorithms fall into two categories: non‐iterative direct^28^ methods perform a reconstruction of the full scan area, usually via Wigner‐distribution deconvolution (WDD)^29^ or single‐sideband ptychography (SSB),^30^ while iterative ones refine it over many iterations, most notably the ptychographic iterative engine^31^ and its later extensions^32^ and improvements.^33^ The latter also include generalised maximum‐likelihood methods that are not only robust with respect to noise and probe aberrations, but also able to correct for errors due to scan positioning and partial coherence.^34^ Iterative methods with defocused illumination allow large areas to be reconstructed at reduced dose,^35^ but using a focused probe is convenient as it allows simultaneous atomically resolved imaging that helps elemental identification via annular dark‐field *Z*‐contrast.^30^ For more information on these algorithms, I refer the reader to recent literature.^26^, ^36^

Here, I analyse convergent‐beam electron diffraction maps recorded with an aberration‐corrected focused probe with a convergence semi‐angle of 34 mrad. The sample is pristine one‐atom thick monolayer graphene, which represents an ideal weak‐phase object where each atom is equivalent and that is impervious to irradiation damage in ultra‐high vacuum at the 60 keV primary beam energy.^37^ The data were acquired on a retractable Dectris ARINA hybrid‐pixel detector^10^ installed on‐axis in a Nion UltraSTEM 100 instrument, whose ultra‐stable sample stage and flexible electron optics make it ideally suited for 4D‐STEM. The high quantum efficiency and dynamic range of the detector, the irradiation stability of the specimen, the low drift of the stage, and the high beam current of up to 200 pA together ensure that these measurements constitute a nearly ideal dataset for benchmarking.

Using these data, I compare a number of open‐source implementations of direct and iterative phase reconstruction algorithms: SSB and WDD, as well as integrated centre of mass (iCOM; the equivalent method is dubbed iterative differential phase contrast in py4DSTEM),^38^ parallax‐corrected bright‐field imaging (i.e. tilt‐corrected bright‐field STEM^39^), and batched iterative gradient descent single‐slice ptychography^36^ (which is essentially equivalent to the widely used ePIE^32^ method, apart from the batching of probe positions which greatly improves convergence^36^). Computational times and the robustness of the reconstruction vary greatly depending on the algorithm and the sampling in both real and reciprocal space, although it should be also acknowledged that different algorithms may be more or less sensitive to small discrepancies of input parameters that are unavoidable in practice.

In the case of the present data, even with high sampling and areal doses of over 10^6^ electrons/Å^2^, phase images show minor variations from the expected uniform atom contrast, partly due to imperfectly corrected residual aberrations, as well as differing sensitivities to reciprocal‐space binning and real‐space sampling, as quantified using a parameter‐based iterative method.^40^ These findings, which include fully open access to both the data and the analysis code, thus provide a useful resource for understanding and further developing electron‐ptychographic methods for quantitative phase imaging.

## 2 RESULTS

### 2.1 Characteristics of the data

Preparation of the pristine monolayer graphene samples, 4D‐STEM data acquisition, and phase reconstruction and quantification are described in the Section 4, with all data and analysis code openly available (see Data and Code). I recorded datasets with two different maximum collected scattering angles controlled by the projector lens settings: a magnified one with 512 × 512 real‐space ***R*** scan positions where the maximum scattering angle in the vertical direction was 36 mrad, and thus the bright‐field disk almost covered the camera, and an extended one with a maximum scattering angle of 109 mrad, with 256 × 256 scan positions. For the magnified setting, I recorded data with the full unbinned sensor with 192 × 192 reciprocal‐space ***Q*** pixels (with the bright‐field disk diameter of ∼ 177 pixels), where the data rate of the 10G fibre‐optic connection to the camera limits pixel dwell time to 33.3 μs, and a 2× hardware‐binned mode with 96 × 96 ***Q*** pixels (bright‐field disk of ∼89 pixels) that allows the fastest possible dwell time of 10 μs per pixel (i.e. 120,000 frames per second). For the extended setting, because of the scattering cross section decreasing as a function of scattering angle, I increased the pixel dwell time to 100 μs, and recorded the full unbinned sensor to ensure good ***Q*** sampling (bright‐field disk of ∼59 pixels).

**FIGURE 1:**
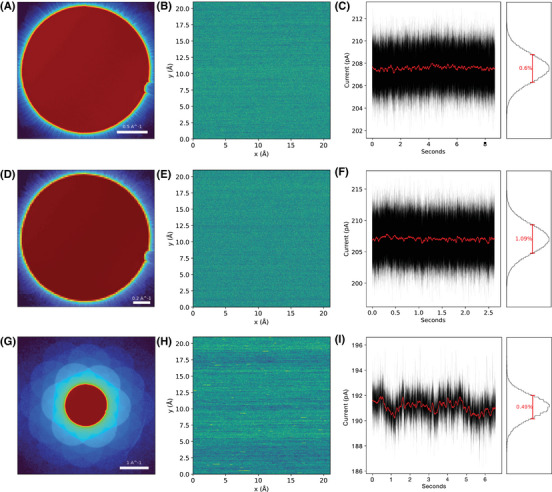
(A) Mean diffraction pattern of the magnified projector lens setting (maximum scattering angle of 36 mrad) unbinned data on a logarithmic intensity scale. (B) Variation of the dose over the 512 × 512 scan pixels (pixel dwell time 33.3 μs). (C) Variation of the current over time. The red curve is a moving average. (D–F) The same plots for the data with 2 × binning and 512 × 512 scan pixels (pixel dwell time 10.0 μs). (G–I) The same plots for the extended projector lens setting (maximum scattering angle of 109 mrad) with 256 × 256 scan pixels (pixel dwell time 100 μs).

The mean convergent‐beam electron diffraction (CBED) patterns recorded over graphene are shown in Figure 1, alongside the spatial and time variation of the dose over the respective 8.8, 2.6, and 6.6 s acquisitions. The standard deviation of recorded current was in the range of 1% as expected for the cold‐field emission gun, with the greatest time variation for the fastest pixel dwell time and with some variation visible in the slow‐scan direction across the horizontal horizontal scan lines. The irradiation doses were counted directly from the diffraction patterns (thus ignoring the negligible scattering outside the detector from such a weakly scattering object) with a correction for the measured detective quantum efficiency of the camera of 0.80 at 60 keV.^10^ The corresponding beam currents for the two datasets were 207, 207 and 192 pA, resulting in respective total doses of 11.4 × 10^9^, 3.4 × 10^9^, and 7.8 × 10^9^ electrons and areal doses of 2.6 × 10^7^, 0.77 × 10^7^, and 1.77 × 10^7^ electrons/Å^2^. These doses are notably larger than typical works on focused‐probe low‐dose reconstructions,^40^, ^41^, ^42^ but as single‐layer graphene is a weakly scattering object, direct comparison of fluences might be misleading. However, comparing the two datasets with different pixel dwell times allows us to assess this directly.

### 2.2 Phase reconstructions

#### 2.2.1 Magnified projector lens setting: software binning in ***Q***

First, I compare phase images reconstructed from the full dataset with 512 × 512 real‐space ***R*** scan positions with the magnified projector lens setting (36 mrad maximum scattering angle) with different degrees of reciprocal‐space ***Q*** binning. Notably, the parallax, SBB and WDD algorithms could not cope with the memory requirements of the full ∼39 GB dataset on our processing workstation with 128 GB of memory (see Section 4), and I will therefore compare binning factors of 2 and above. The uncompressed data array sizes alongside reconstruction times for different algorithms are shown in Table 1. In terms of computational timings on this hardware, all algorithms perform significantly faster when data is binned in **Q**, although there seems to be little further benefit from increasing the bin factor from 16 to 32. Comparing the speed of the algorithms is caveat by the fact that I used greater ***Q***‐space padding for the iterative gradient descent when memory allowed and made use of GPU acceleration, and needed to vary the number of alignment iterations for parallax to achieve optimal results. In general, aberrations needed to be fit up to the fifth order – we noticed that third‐order software correction on a fifth‐order hardware‐corrected instrument often resulted in worse phase uniformity than using no aberration correction. For some reconstructions, fitting seemingly did not work very well in this semi‐atomated workflow, as phase uniformity did not improve or even worsened.

**TABLE 1:** Data sizes (in megabytes) and computational timings (in seconds) for different phase reconstruction algorithms for the full 512 × 512 real‐space scan positions (**R** px) with different degrees of reciprocal‐space binning resulting in different numbers of pixels (***Q*** px) in the convergent‐beam electron diffraction patterns (CBED). The listed algorithms are integrated centre of mass (iCOM), parallax‐corrected bright‐field (parallax), iterative gradient descent (iter. GD), single‐sideband (SSB) and Wigner distribution deconvolution without and with aberration fitting (AC).

Scan (***R*** px)	Bin in ***Q***	CBED (***Q*** px)	Array (MB)	iCOM (s)	Parallax (s)	Iter. GD (s)	SSB (s)	SSB (AC) (s)	WDD (s)	WDD (AC) (s)
512 × 512	2	96 × 96	9664	10	—	1198	365	390	378	382
	4	48 × 48	2416	8.8	157	698	113	118	156	158
	8	24 × 24	604	8.1	153	242	43	48	44	44
	16	12 × 12	151	8.0	16	135	25	29	26	27
	32	6 × 6	38	7.6	19	108	20	23	21	20

**FIGURE 2:**
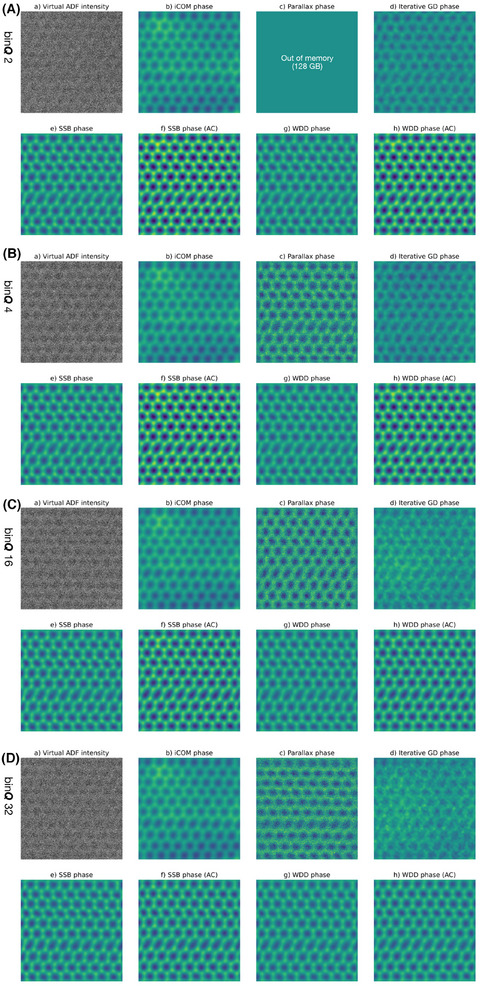
Comparison of the effect of ***Q***‐binning on phase images of the 36 mrad maximum scattering angle dataset recorded with a pixel dwell time of 33.3 μs over pristine monolayer graphene reconstructed with the different algorithms indicated over each panel. (A) Images for a ***Q***‐binning factor of 2, (B) for 4, (C) for 16, and (D) for 32. The field of view is 2.1 × 2.1 nm^2^ and the phase intensity scale ranges from –15 to 15 mrad.

Visually, the phase images look similar across the range of binning factors considered here. Specifically, there is little change between 2× and 16× binning for this magnified setting that is optimal for bright‐field ptychography except for the aberration‐corrected SSB and WDD, where contrast is noticably increased. I therefore display in Figure 2 only the phase images reconstructed from the 2×, 4×, 16× and 32× binned data for each of the algorithms, alongside a virtual annular dark‐field (ADF) image with the inner radius set just outside the bright‐field disk. Such a large magnification of the diffraction patterns has the benefit of maximising the collected bright‐field signal, but is obviously disadvantageous for virtual ADF imaging. Notably, a distortion likely due to small stage jump is visible as a vertical ‘stretching’ about one third of the way down the 2.1 × 2.1 nm^2^ field of view. The atomic phase variation is quantified in Section 2.3; importantly, the phase optimisation method I use is able to at least partially account for such distortions.

Remarkably, although the parallax method nominally requires bright‐field contrast to align the pixels corresponding to different scattering vectors, I found parameters that resulted in very good results even for such a weakly scattering object. Important here was to allow only a few (between one and three, typically) alignment iterations at the largest possible binning (typically 32). Further, while the iterative gradient descent algorithm is typically intended for defocused‐probe reconstructions, with a judicious selection of parameters also it was also able to perform rather well here albeit at a lower resolution due to the limited ***Q*** *‐*space padding allowed by memory – as we will see below, in some situations it may even outperform other algorithms.

Regarding phase uniformity, it is clear that iCOM, and to a smaller extent iterative gradient descent, exhibit greater low‐frequency phase variation than the other methods. The signal‐to‐noise ratio in iCOM is known to suffer at small spatial frequencies ***k***, with an analytical expression inversely proportional to ***k***.^36^ For iterative GD, the reason is analytically less clear, but it appears empirically that small spatial frequencies are the hardest to converge, and the exclusion of larger scattering angles by the magnified projector lens setting is further disadvantageous for this algorithm. Note also that although it would easily improve phase uniformity, for unbiased comparisons I did not here apply any smoothing or high‐pass filtering (which would be directly supported within the reconstruction algorithms in py4DSTEM).

#### 2.2.2 Fastest acquisition hardware‐binned data

**FIGURE 3:**
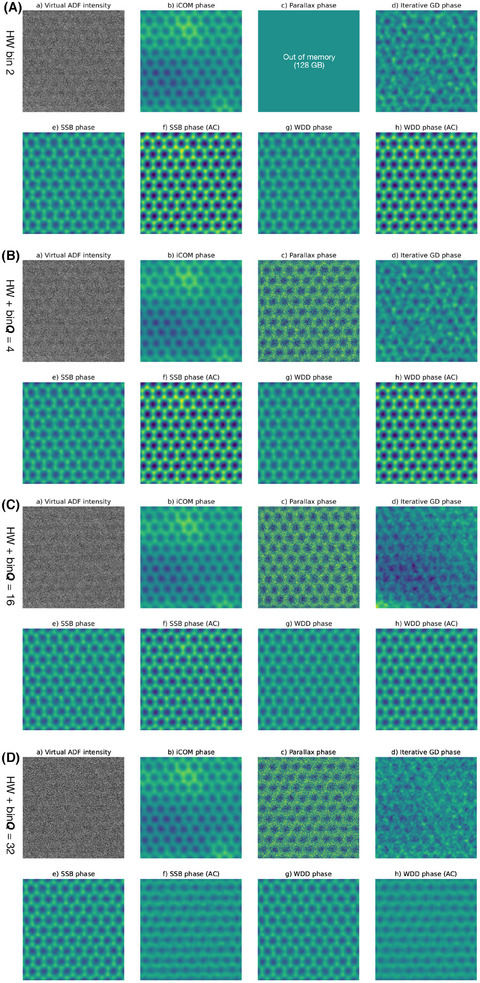
Comparison of the effect of ***Q***‐binning on phase images of the 36 mrad maximum scattering angle dataset recorded with pixel dwell time of 10.0 μs over pristine monolayer graphene reconstructed with the different algorithms indicated over each panel. (A) Images for only 2× hardware binning, (B) for a further software ***Q***‐binning factor of 2 (total 4), (C) for 8 (total 16), and (D) for 16 (total 32). The field of view is 2.1 × 2.1 nm^2^ and the phase intensity scale ranges from –15 to 15 mrad.

With such a large bright‐field disk recorded on the camera, significant binning is possible and even desirable. Before I discuss real‐space thinning, let us thus first consider a separate dataset collected with the 2× hardware‐binning required to run the ARINA detector at its maximum frame rate of 120,000 fps. Although the beam current was still very high at 207 pA, the shorter pixel dwell time resulted in three times lower doses than in the full dataset. Again, the phase images look similar across the range of binning factors, so I display in Figure 3 only the phase images reconstructed from only the hardware binning, and additional 2×, 8× and 16× software‐binned data for each of the algorithms. The distortion due to the stage shift seen in the previous dataset is notably not present here.

Although broadly speaking the results are similar to the full data in Figure 2, with rather high additional binning still being possible, some interesting differences can be discerned. The iterative gradient descent method cleraly struggles with this data, presumably due to the lower dose that was collected, but parallax remains robust throughout. Remarkably, although aberration correction becomes poor at the highest binning, naive SSB and WDD retain atomic resolution even for the very highest total binning factor of 32, which corresponds to diffraction patterns with only 6 × 6 pixels. Clearly data sizes can be significantly reduced by binning, as has also been noted before, but such a large projector lens magnification optimises this further. It is unfortunate the ARINA is not able to bin by more than a factor of two in hardware, since greater binning would further reduce the data rate and help unleash the true maximum speed of the underlying electron counting application‐specific integrated circuit of the detector.^43^

#### 2.2.3 Magnified projector lens setting: software thinning in ***R***

**FIGURE 4:**
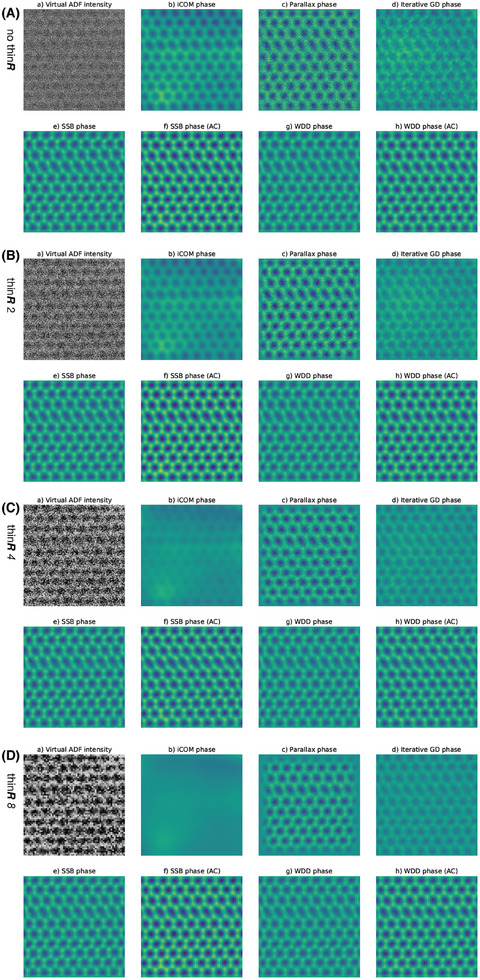
Comparison of the effect of ***R***‐thinning on phase images of the 36 mrad maximum scattering angle dataset recorded over pristine monolayer graphene reconstructed with the different algorithms indicated over each panel. A *Q*‐binning factor of 16 was used throughout. (A) shows images for no thinning, (B) for a R thinning factor of 2, (C) for 4, and (D) for 8. The field of view is 2.1 × 2.1 nm^2^ and the phase intensity scale ranges from –15 to 15 mrad.

Before considering the extended projected lens setting, let us first investigate the effect of thinning the full data in ***R***, that is, omitting every *n*th scan pixel, which both reduces the sampling and the total dose available for the reconstructions. The real‐space sampling of the original data was 0.041 Å/pixel, corresponding to a relative probe overlap (defined as the linear distance offset) of 96% (assuming a round probe with full‐width at half‐maximum of 1.1 Å), which reduces to 70% for a thinning factor of 8 (real‐space sampling of 0.33 Å/pixel), just above the criterion proposed by Bunk.^44^ For the comparisons shown in Figure 4, I again used the full 192 × 192 ***Q*** px data acquired with a dwell time of 33.3 μs per pixel with a Q‐binning factor of 16 (note that for ease of comparison, the top‐most images labeled (a) thus reproduce the images labeled (c) in Figure2). The difference to *Q*‐binning is striking for iCOM, with atomic contrast being entirely lost for thinning factor 8. However, the other algorithms are still able to retain good resolution, with iterative gradient descent even benefitting from the reduced sampling.

**FIGURE 5:**
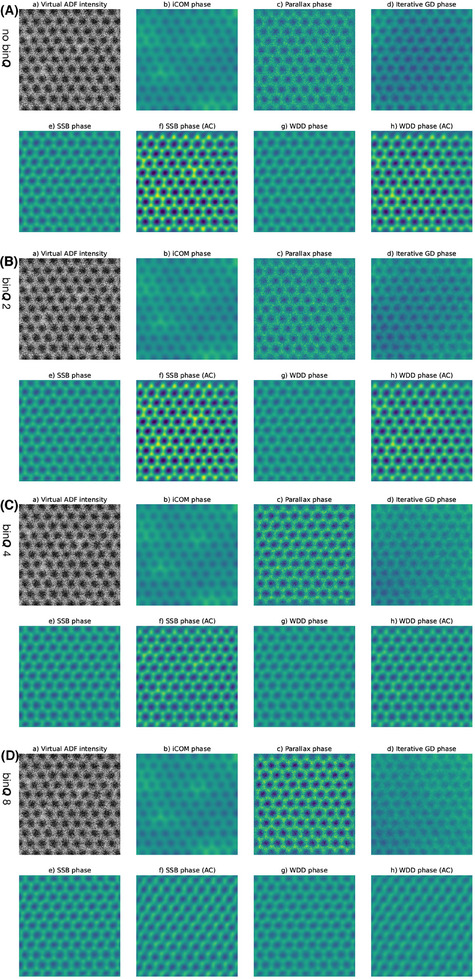
Comparison of the effect of ***Q***‐binning on phase images of the 109 mrad maximum scattering angle dataset recorded with a pixel dwell time of 100 μs over pristine monolayer graphene reconstructed with the different algorithms indicated over each panel. (A) Images for no binning, (B) for a ***Q***‐binning factor of 2, (C) for 4, and (D) for 8. The field of view is 2.1 × 2.1 nm^2^ and the phase intensity scale ranges from –15 to 15 mrad.

#### 2.2.4 Extended projector lens setting: software binning in Q

Phase reconstructions of the data recorded with the extended projector lens setting with 109 mrad maximum recorded scattering angle with 256 × 256 real‐space *R* scan positions with different degrees of reciprocal‐space *Q* binning are compared in Figure 5. Although the overall visual impression is similar to the magnified setting images despite this data including fewer scan positions (corresponding to thinning factor 2 with respect to the magnified data), a few differences can be easily noted. Unsurprisingly, the virtual ADF images have much better contrast now that larger scattering angles are included. Iterative gradient descent reconstructions now show a visibly greater spatial resolution for most binnings, and parallax continues to perform well. The direct ptychography algorithms also do not fare too poorly enve when sampling of the bright‐field disk decreases to below 8 pixels for the binning factor of 8, although fitting aberrations correctly clearly becomes increasingly difficult. However, naive SSB and WDD still perform remarkably well, suggesting extended projector lens settings with moderate binning may provide a good compromise when good virtual ADF imaging is desirable and both direct and iterative methods are used. However, some differences in the phase variation can be noted, as will be discussed next in section 2.3.

### 2.3 Quantification of phase variation

Finally, let us turn to the quantification of the atomic phase shifts from the reconstructed phase images. Due to the complicated nature of the contrast transfer function of SSB^25^ (specifically, atomic phase contrast has a negative halo which can influence the phase at neighboring sites^40^) and other ptychography methods,^26^ and due to the effect of scan distortions, drift, or sample tilt, simple methods based on, for example, Gaussian fitting or Voronoi integration may not give reliable results. I therefore used the parameter‐based iterative method developed by Hofer and Pennycook^40^ as described in Section 4.

Briefly, in this optimisation method, an initial atomic model is created to correspond to the visible part of the lattice. This is then converted to a point potential based on the model, after which the contrast transfer function (CTF) of a given ptychography method is converted to the point‐spread function applied to each position, resulting in a phase image matching the model positions and intensities. The model is then iteratively optimised so that the correlation between the model image and the experimental phase image is maximised. Notably, this optimises not only the positions of the atoms in the model, but also the strengths of the point potential at each location, from which I then derive the atomic phase shifts – and, crucially for this study – their variation over the lattice. The means of the distributions of atomic phase values estimate the absolute phase magnitude for each method, while their variation estimates how well each algorithm copes with noise and aberrations.

As an improvement of the original phase optimisation method,^40^ the CTFs of iCOM, parallax and iterative gradient descent method^36^ are also now explicitly included. One caveat should however be noted: the convergence of the method currently requires the handcrafting of a relatively accurate model for the field of view, which changes not only between acquisitions, but may vary slightly depending on the exact reconstruction algorithm. This led to the fitting occasionally failing, necessitating a tedious manual verification and modification of the model. Clearly, a better approach in the future will be to use machine vision to detect the lattice and to create the model automatically to match each phase image.

**FIGURE 6:**
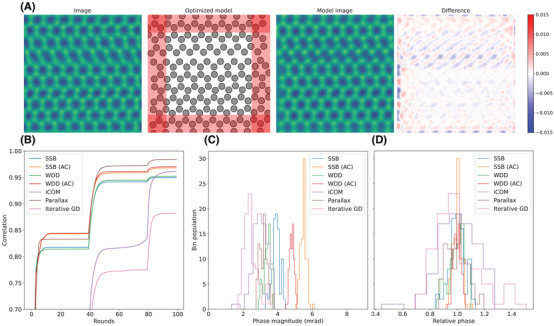
Example of atomic phase quantification for the full magnified projector lens setting dataset with a ***Q***‐binning factor of 16. (A) The experimental SSB phase image, the optimised atomic model matching the field of view after a three‐stage optimisation procedure with 110 rounds in total (see Section 4), the simulated model image, and its difference to the experimental one (colourmap ‘bwr'). The phase intensity colour scale spans from –15 to 15 mrad. (B) Correlation between experimental and optimised model images as a function of iteration rounds for all reconstruction algorithms. (C, D) Distributions of the absolute (C) and relative (D) optimised atomic phase shifts (with atoms within 15% of the edge excluded as shown in red on the atomic model in panel (A) for all reconstruction algorithms.

Figure 6 shows examples of the procedure for the full magnified projector lens setting dataset with a ***Q***‐binning factor of 16. This is a particularly challenging case due to the scan distortion, but as we can see, the iterative optimisation procedure is able to satisfactorily though not perfectly account for the imperfections of the shown SSB image (panel a). For parallax, the experimental phase images contained much more shot noise that led to spuriously low correlations, so these were smeared with a Gaussian of width 0.25 Å before optimisation. The convergence of the correlations for each method are shown in panel b; I note that the somewhat low values are partly explained by the edges, but the distortion does contribute. As contrast at the edges was not correctly reproduced, 15% of the field of view from each side was always omitted from the further phase quantification. In panel c, histograms corresponding to the absolute atomic phase magnitudes are shown for each of the methods (with panel d showing the corresponding relative spread).

**FIGURE 7:**
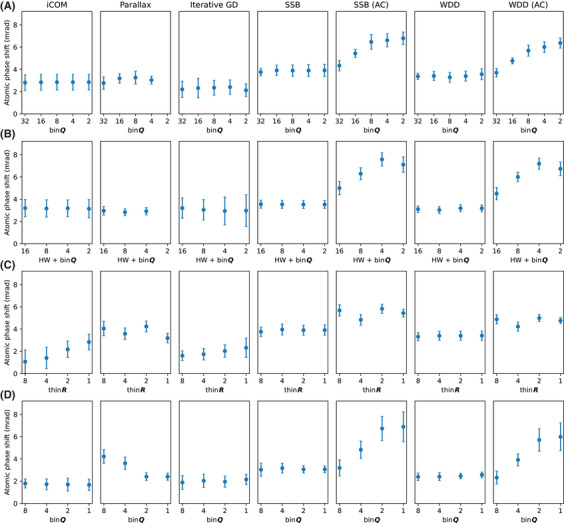
Quantification of the atomic phases for each of the reconstruction algorithms for different datasets and levels of binning and thinning. The points show mean phases with 95% confidence intervals based on their standard error. (A) Magnified projector lens setting full dataset ***Q***‐binning. (B) Magnified projector lens setting fast dataset ***Q***‐binning. (C) Magnified projector lens setting full dataset ***R***‐thinning. (D) Extended projector lens setting ***Q***‐binning.

The quantification is presented in Figure 7 for all binning and thinning values including the few that were omitted from the images shown in Figures 2, 3, 4, 5. Overall, the absolute values of the atomic phase shifts are in relatively good agreement, with SSB and WDD especially for the less binned data with fitted aberration correction showing slightly larger values. In terms of the phase variation, iCOM (not to mention the ADF images that were not quantified) and parallax appear to perform somewhat more poorly than SSB or WDD, whereas iterative gradient descent produces slightly greater variation with the magnified projector lens setting and especially for the faster dataset, but performs quite well in comparison for the data thinned in ***R*** as well as the extended setting. Comparison of the full and fast magnified setting data for SSB and WDD with a threefold difference in dose suggests that these reconstructions are not fluence‐limited, as the variation of phase is even smaller for the lower‐dose data. The unexpected larger variation of the aberration‐corrected full data where a sample shift was present (visually apparent in the top panels of Figure 2) may indicate that this distortion has interefered with the accurate fitting of the aberration coefficients.

#### 2.3.1 Dependence of WDD phase on ε

Finally, the dependence of the WDD phase values on the Wiener filtering parameter ε requires a separate discussion. The original theory^29^ and its algorithmic implementations^45^ include an additive parameter ε in the denominator of the Wigner distribution term. This is meant to act as a Wiener filter, and its value should be small and presumably is not meant to influence the results significantly. I originally found that a suggested default value of 0.01 produced poor‐quality phase images, and thus settled on the smallest value that did at 0.05. However, upon further inspection it turned out that the WDD phase magnitudes converge towards the SSB values when ε *increases* towards 1.

**FIGURE 8:**
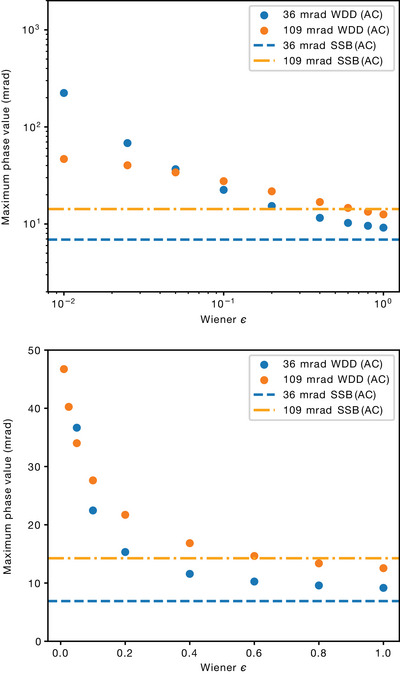
Convergence of the aberration‐corrected WDD maximum phase value as a function of increasing Wiener ε parameter compared to respective SSB value for two datasets: full magnified projector lens setting data binned by 4 in ***Q***, and the unbinned extended projector lens setting data. (A) A log‐log scale plot showing the full variation. (B) A linear scale with the largest WDD phase values at small ε cropped.

To quantify this, I reconstructed selected datasets (using iteratively fitted aberration coefficients, though the results are similar without) with different values of ε and, for simplicity and to show this effect is not dependent on the CTF‐based optimisation, compared the maximum phase values to SSB reconstructions of the same data. The results are shown in Figure 8. The convergence of the maximum phase values as ε increases is clearly apparent, though there still seems to be small discrepancy compared to the respective SSB phase values. Such large values of ε appear to go against the original intention behind the algorithm, suggesting that the original theoretical derivation may need revisiting due to the effect the Wiener filter has on realistic experimental data (please also see Section 4.3 for discussion of replication of the findings with an independent implementation).

## 3 CONCLUSIONS

To summarise, I have used graphene as an ideal uniform phase object to compare direct and iterative electron‐ptychographic phase reconstruction algorithms with data collected at high electron dose in an effort to benchmark the algorithms’ performance and reliability. The electron optics were aligned with a maximally magnified projector lens setting for optimal sampling of the bright‐field disk, and I compare that to an extended projector lens setting typically used for defocused‐probe data collection and super‐resolution. I have evaluated the algorithms' computational efficiency and tolerance to reciprocal‐space binning and real‐space thinning of the data, and quantified the atomic phase shifts to provide guidance for the sampling required for desired levels of relative phase precision with each method. Although the methods mostly agree, iterative gradient descent clearly benefits from collecting larger scattering angles, whereas direct methods are faster and can operate reliably with sampling only a few dozen pixels of the bright‐field disk. My findings also suggest that the Wigner distribution deconvolution phase values sensitively depend on the Wiener filter parameter in a way that appears to go against the original intention of the algorithm. By using exclusively open‐source software tools and sharing my data and code openly, I hope this contribution helps spur further development and standardisation of electron ptychography as a truly quantitative technique for the detection of electrostatic potentials, charge transfer, magnetism, and beyond.

## 4 METHODS

### 4.1 Sample preparation

Commercial monolayer graphene grown by chemical vapour deposition and transferred by the vendor onto TEM grids with Quantifoil carbon foil supported on an Au mesh from Graphenea were used. Clean and defect‐free (pristine) monolayer regions were identified by imaging and the 4D‐STEM data subsequently recorded. The scanned area was set to approximately 2.1 × 2.1 nm^2^, as with the lattice constant of graphene this conveniently produced a nearly periodic field of view.

### 4.2 4D‐STEM data acquisition

Data was recorded using the Nion Swift software^46^ (version 0.16.10), acquired on the Nion UltraSTEM 100 aberration‐corrected STEM instrument operated at 60 keV with a probe convergence semi‐angle of 34 mrad as verified from the averaged convergent‐beam electron diffraction pattern and instrument parameters, and the Dectris ARINA direct‐electron detector (with Si sensor material). The beam current was close to 200 pA for all collected datasets, and the sample was at room temperature in ultra‐high vacuum (near 10^−9^ mbar).

Different magnifications were set up using the projector lens system of the microscope, which I denoted by the maximum scattering angle captured by the detector array in the vertical and horizontal directions (diagonal scattering angles were correspondingly larger), namely 36 mrad (∼ α) and 109 mrad (∼3α). This allowed us to study the effect of the experimental projector lens setting on the reconstruction performance, ranging from maximal sampling of the bright‐field disk to a setting more conducive to iterative algorithms and super‐resolution images (the latter of which was explicitly not my aim here).

Most data was collected using the full unbinned 192 × 192 pixel (***Q*** px) array and binned to varying degrees in software to study the tolerance of the algorithms to limited reciprocal‐space sampling, with a pixel dwell time of 33.3 μs, but some data used 2× hardware binning to 96 × 96 pixels to reach the highest possible frame rate with pixel dwell time of 10 μs. For the magnified projector lens setting scan, I used 512 × 512 pixels for sufficiently high sampling, which was thinned in software by omitting scan positions to study the effect of limited real‐space sampling. For the extended projector lens setting, I used fewer 256 × 256 scan pixels to keep the total acquisition time roughly constant with the longer 100 μs pixel dwell time used to increase signal for larger scattering angles. For the purposes of the iterative gradient descent reconstructions, the probe was separately recorded over vacuum for each setting.

### 4.3 Phase reconstructions

All ptychographic phase reconstructions were performed using the py4DSTEM package^36^, ^38^ (version 0.14.19). For SSB and WDD, apart from the experimental settings – probe convergence semi‐angle, primary beam energy, scan‐step size, and ***Q***–***R*** rotation (see below) – for WDD the only other adjusted input parameter was the small positive constant ε introduced to avoid dividing by zero values in the Wigner deconvolution. I initially used a value of 0.05 for this constant, but as discussed in Section 2.3, I found that counter‐intuitively, the larger this value, the smaller were the absolute phase magnitudes and the more in line with the other methods they were. I therefore settled on a value of to 1.0 to achieve converged phase values for the results presented here.

When aberration correction was enabled for SSB and WDD, the aberration coefficients were recursively fitted up to 5th radial order based on five sets of double‐disk overlaps. However, it should be noted that not doing aberration correction merely assumes the values of all coefficients to be zero without providing any advantage in terms of simulation time. In early stages of this work presented at conferences,^47^, ^48^ I have used the PyPtychoSTEM package^45^ instead, which produced similar results. PyPtychoSTEM did require somewhat less memory but produced less reliable reconstructions especially when fitting aberrations, though the computational speed of SSB without aberration correction was also significantly faster. Notably, the same sensitivity of the WDD phase magnitudes to the value of ε was present with that code. This suggest to me that this is not an issue with the implementations, but rather an unexpected and frankly undesirable feature of the original theory, at least when applied to realistic electron‐ptychographic data.

For the iterative reconstructions, the py4DSTEM iterative differential phase contrast method (essentially identical to integrated centre of mass, which is how I label it here to avoid confusion) was used to verify the rotation angle between the scan and the reciprocal‐space directions (***Q***–***R*** rotation) determined by the camera orientation – these were set to be as close to aligned as possible when the electron‐optical setup was made – resulting in an angle of typically 3 degrees found by minimising the curl of the x‐y centre‐of‐mass (COM) signals. Notably, I found that blurring these with a Gaussian of 4 px greatly improved the robustness of finding the angle, and that 20 iterations of the algorithm was more than sufficient for convergence. Importantly, I uncovered a factor of 2π difference between this and the other ptychography methods, which I have corrected by hand in all of my analyses (pending code revision by the py4DSTEM developers).

For parallax‐corrected bright‐field imaging, an intensity threshold of 0.65 for detecting the bright‐field disk pixels was used, with the ***Q***–***R*** rotation forced to the previously determined value. Only the coarsest possible binning (bin value of 32, or the closest to it if the data was binned more) and between 1 and 5 iterations was used to align the different tilts; crucially, I found that any further iterations or finer binning resulted in poor subpixel alignment and contrast transfer function fitting, and thus resulting phase images, presumably due to the extremely low bright‐field signal level from a lattice consisting of a single layer of light elements.

Finally, for batched iterative gradient descent single‐slice ptychography, the ***Q***–***R*** rotation was again forced, the object type was set to ‘potential’ and object positivity set to False (to treat the specimen potential as a pure weak phase object), and the probe was allowed to update (as fixing it did not result in good reconstruction results). The object was padded by 12 pixels in both directions, and the diffraction‐space padded by a factor of 3 or 2 times whenever memory allowed to improve the resolution of the reconstruction for the magnified projector lens setting. Separately recorded vacuum probes were provided to the algorithm to reduce probe‐object mixing. The batch size was set to 512 for optimal memory use, and 15 iterations were found to be sufficient for convergence, with more resulting in more severe mixing in my testing. Other reconstruction parameters were at the default settings, including the update step size of 0.5 and a weight of the maximum probe overlap intensity of 1. More information can be found in Ref. [36].

All reconstructions were performed on a 32‐Core AMD Ryzen Threadripper 1950X (parallelised over workers to different degrees depending on the algorithm), except for the batched iterative gradient descent, which was accelerated using CuPy on an NVidia RTX4090 GPU (with data stored in the CPU RAM so that sufficient memory was available for all datasets). Parallax would also benefit greatly from GPU acceleration, but memory usage became a bottleneck as CPU RAM data storage was not available there. However, the relative performance of the algorithms may be different on hardware with different constraints (single‐core performance vs. number of cores, memory size vs. memory bandwidth, and so on). Further, it needs to be acknowledged that these timings represent a specific implementation of these algorithms on specific hardware, and so broader conclusions on the inherent limitations of the algorithms themselves must be drawn only with care.

### 4.4 Phase quantification

The atomic phase shifts were determined by parameter‐based iterative optimisation^40^ (commit 8860c37e of the ‘newctfs’ branch of the forked repository at the author's GitLab^50^). The optimizations were based on an atomic model, which was a 9 × 5 orthogonal supercell of graphene that was manually translated, wrapped and occasionally cropped and scaled to roughly match the fields of view of each dataset. Each experimental phase image was binned by 4 for speed (except for the iterative gradient descent images, which had a lower resolution, and the data thinned in ***R*** which had fewer pixels), and the model was iteratively modified to maximise the correlation between a phase image simulated with the contrast transfer functions of each ptychography method with scattering potentials placed at the atomic positions of the model and the experimental images. Optimisation proceeded in three stages: 40 rounds of quick optimisation matched the field of view, translation and scale (and blur, which was not used), then 40 further rounds matched also the positions of the model atoms, and finally 20–80 rounds optimised also their potential strengths. These then yielded the atomic potential strengths corresponding to the atomic phase shifts, which were binned into scistograms to quantify the phase variation after atoms within 15% of the edges of the field of view were omitted. The mean (based on the bin centres) and standard error of the mean (at 95% confidence level) of the phase variation were additionally calculated for plotting.

## CONFLICT OF INTEREST STATEMENT

The author declares no conflicts of interest.

## DATA AND CODE

The 4D‐STEM datasets stored as calibrated py4DSTEM DataCube objects used in this article are openly available on the University of Vienna Phaidra repository at https://doi.org/10.25365/phaidra.564. All of the analysis code is openly available on the author's GitHub repository at https://github.com/TomaSusi/arina‐ptycho. All of software packages used in the analyses are open‐source Python codes that are available on their respective GitHub or GitLab pages.
